# Draft Genome Sequence of the First Isolate of Extensively Drug-Resistant *Mycobacterium tuberculosis* in New Zealand

**DOI:** 10.1128/genomeA.00319-14

**Published:** 2014-05-08

**Authors:** Ronan F. O’Toole, Bushra M. Johari, Micheál Mac Aogáin, Thomas R. Rogers, James E. Bower, Indira Basu, Joshua T. Freeman

**Affiliations:** a Department of Clinical Microbiology, School of Medicine, Trinity College Dublin, St. James’s Hospital, Dublin, Ireland; b LabPlus, Auckland City Hospital, Auckland, New Zealand; c School of Molecular Medicine and Pathology, Faculty of Medical and Health Sciences, University of Auckland, Auckland, New Zealand

## Abstract

Extensively drug-resistant (XDR) tuberculosis has now been described in >90 countries worldwide. The first case of XDR tuberculosis (XDR-TB) in New Zealand was recorded in 2010. We report the draft whole-genome sequence of the New Zealand isolate, NZXDR1, and describe a number of single-nucleotide polymorphisms that relate to drug resistance.

## GENOME ANNOUNCEMENT

Tuberculosis (TB) is a leading cause of infectious mortality worldwide, killing approximately 1.3 million people each year (1). Resistant forms of the *Mycobacterium tuberculosis* complex (MTBC) threaten the global management of TB, with an estimated 450,000 cases being multidrug resistant (MDR), defined as resistant to rifampin and isoniazid (1). A subset of these cases, approximately 10%, is also resistant to the second-line drug classes, fluoroquinolones, and injectable aminoglycosides and is referred to as extensively drug resistant (XDR) (2). Resistance in XDR strains is often not confined to the above TB drug classes (3, 4). Hence, successful treatment is dependent upon complete knowledge of the drug susceptibility profile of each isolate.

Phenotypic drug susceptibility determination for XDR isolates can take a number of months from the date of patient specimen collection to complete and, in some cases, occurs following failure of an MDR treatment regimen. Improvements in diagnostic technologies are required to enable rapid early identification of drug-susceptible, MDR-, and XDR-TB, leading to the optimal selection of anti-TB drugs and minimization of further transmission of resistant strains.

Here, we applied next-generation sequencing (NGS) for whole-genome analysis of drug resistance mutations in the first clinical isolate of XDR *M. tuberculosis* in New Zealand from 2010 (5). The genomic DNA of the isolate (strain NZXDR1), which belongs to lineage 2 (East Asian) (6), was sequenced using an Illumina MiSeq instrument. A total of 2,331,938 paired-end reads were mapped to the *M. tuberculosis* strain H37Rv reference genome (accession no. AL123456.3) by Burrows-Wheeler Alignment (7). This yielded an average read depth of 31-fold, covering 98.5% of the H37Rv genome. A consensus sequence was called using the SAMtools software (8), producing a 4,224,610-bp draft assembly of 124 contigs. Single-nucleotide polymorphism (SNP) analysis was performed using Geneious R7, and a total of 1,365 SNPs were detected in the assembled NZXDR1 genome with respect to H37Rv, of which 747 were nonsynonymous.

Nonsynonymous mutations were identified in the genes Rv0667 (*rpoB*) (S450L) and Rv1908c (*katG*) (S315T and R463L). There is strong correlation between *rpoB* S450L (*rpoB* S531L in *Escherichia coli*) and *katG* S315T substitutions with resistance to the first-line drugs rifampin and isoniazid, respectively (4, 9). Mutations were also detected in the Rv3795 (*embB*) gene (M306I and G603R), which is associated with resistance to another first-line drug, ethambutol. Regarding second-line drug resistance, SNPs were identified in the genes Rv0006 (*gyrA*) (E21Q, D94A, S95T, and G668D) and MTB000019 (*rrs*) (A1401G), which are related to resistance to fluoroquinolones and aminoglycosides, respectively, as well as in the Rv0682 (*rpsL*) and Rv3854c (*ethA*) genes, associated with streptomycin and ethionamide resistance, respectively. Not all of the identified mutations were detected in previous line-probe assay analysis (5), and earlier resistance detection for streptomycin and ethionamide was reliant on culture-based assays. Furthermore, from the genome sequence, resistance mutations were detected in the *mmaA2* and *nudC* genes, which are linked to thioacetazone resistance (4). This underscores the higher resolution provided by NGS in drug resistance detection and the potential benefits the technology offers medical laboratory diagnostics.

### Nucleotide sequence accession number.

This whole-genome shotgun project has been deposited at DDBJ/EMBL/GenBank under the accession no. CCBK000000000.
